# Editorial

**Published:** 2008-10

**Authors:** Mukund Thatte

**Affiliations:** Editor, Indian Journal of Plastic Surgery, 402 Vimal Smruti, 770 Dr. Ghanti Road, Dadar, Mumbai - 400 014, Maharashtra, India. E-mail: mthatte@vsnl.com

I have great pleasure in placing before you this theme issue on aesthetic surgery. It has been very ably steered by Dr. K. Ramachandran as the Guest Editor and our Deputy Editor Dr. Milind Wagh deserves special mention in this particular endeavour for his considerable help.

Aesthetic surgery remained the step child of Indian Plastic surgery for a very long time, for various reasons, both social as well as cultural. The old argument that resources cannot be allocated to aesthetic surgery when burn contractures are waiting just does not wash anymore. If resources are falling short then they need to be augmented, this is the way supply side management ought to work, not by restricting sub specialties. Today I think the question is not whether Plastic surgeons should be doing it but rather how can they do it with finesse, high level expertise and ethically.

Aesthetic surgery is a response to the basic human desire to ‘look good’ in a given social and cultural milieu. In the past, there was this faint whiff of the slightly less than desirable about doing it. Those doing exclusive reconstructive work felt this was pandering to vanity. I think it is neither of the above. It is a need felt by millions and unless completely unrealistic goals are set by the patient, it is not our job to be judgemental. It took me a long time to realise that some of the giants in reconstructive sub specialties had a very good aesthetic practice by the side. In fact, they probably could afford to do the other work at vastly reduced compensation levels (especially compared to the complexity of the microsurgery etc.) because they had this other practice on the side to augment their incomes. In the IPRAS congress in New Delhi in 1987, Sir Benjamin Rank had said: ‘to specialise in hand surgery means to do 90% hand surgery and 10% other things for your practice.’ I think he was probably referring to something similar. The world around us is changing, whatever our thoughts, the social need for aesthetic surgery is growing, and unless we change ourselves in response to it we will be left behind. Not only will inadequately qualified people fill in the ‘space’ but eventually we will no longer be identified as the proper professionals to do it. This outcome can only be avoided by teaching and training, and this issue is an effort to fill in the void in formal education.

This issue is a theme issue and has invited articles from authorities in the field, both from this country as well as from overseas. The articles are somewhat didactic in nature by design. They are intended for the trainees as well as for those not adequately exposed to aesthetic surgery. The idea is to give a comprehensive overview of the subjects and useful knowledge to hopefully reduce the ‘learning curve’. It cannot replace hands on teaching or learning, but a beginning can be certainly made.

The next step ought to be the appointment of visiting consultants in all teaching units to augment the sub specialty spectrum of each unit. This can be outside the MCI full time guidelines, they need not be ‘recognised’ teachers, they will merely enhance the skill sets available to the Department chiefs, this way there are no recognition hassles but the trainees still get good hands on experience. Finally, some of the active aesthetic units in the country can start certification courses and apply to the DNB programme and MCI for validation. If we can have a FNB in hand surgery then why not in aesthetic surgery? I hope unit heads will read this and act. The rest as they say is in the hands of the almighty.

Best wishes for the festive season.

